# The Teacher’s Role in Preventing Bullying

**DOI:** 10.3389/fpsyg.2019.01830

**Published:** 2019-08-14

**Authors:** Lisa De Luca, Annalaura Nocentini, Ersilia Menesini

**Affiliations:** Department of Education, Languages, Intercultures, Literatures and Psychology, University of Florence, Florence, Italy

**Keywords:** bullying, victimization, teachers, teacher’s competence, self-efficacy, job satisfaction, teacher’s response

## Abstract

The teacher plays an important role in the management of classroom bullying (Yoon and Bauman, 2014). Therefore, understanding and fostering teachers’ characteristics able to predict successful responses to bullying and victimization is a priority for prevention programs. The aim of this study was to evaluate whether the association between the teacher’s individual characteristics, such as her/his competence in regard to the phenomenon, job satisfaction, and self-efficacy, and the school level of bullying/victimization was mediated by the teacher’s intervention when an episode of bullying occurred. The study included 120 teachers (17.5% boys; 79.2% girls), between the ages of 25 and 66 (mean age = 48.21; SD = 9.22), and 1,056 students (40.3% boys; 59.6% girls), between the ages of 11 and 17 (mean age = 13.09; SD = 1.46). A total of 57% of the students were attending secondary middle school and 42.2% were in secondary high school. Path analyses showed that for perpetrated behaviors, teachers’ competence on bullying affects students report of bullying through a higher likelihood of teachers’ intervention after a bullying episode occurred. The indirect effect resulted significant. Lower levels of bullying and victimization were associated with teacher job satisfaction, thus indicating how professional fulfillment can influence the classroom climate. The model for victimization was the same, except that the indirect path was not significant. Findings are discussed in terms of teachers’ involvement in bullying intervention and prevention.

## Introduction

Teachers are in an influential position as educators and agents of socialization, helping to promote healthy relationships among students and to prevent negative interactions (Smith et al., 2004). Teachers are often present when an episode of bullying occurs, and they are often the first adults that students contact (Wachs et al., 2019). Teachers could react in a number of ways after a bullying episode, including intervening, observing the situation, not intervening, ignoring and trivializing the bullying (Rigby, 2014). They can monitor bullying incidents, they might intervene in support of the victim or the bully, and/or they can discuss the relevance of a positive class climate with the group. Students expect teachers to actively intervene when bullying occurs (Crothers et al., 2006; Crothers and Kolbert, 2008; Rigby, 2014), although in some cases teachers are unaware of children’s victimization experiences and are viewed by children as providing limited support to the victims (Fekkes et al., 2005; Haataja et al., 2016). Very few studies examine how teachers intervene in bullying situations, and even less analyze the impact of those interventions (James et al., 2008; Merrell et al., 2008; Espelage et al., 2012; Menesini and Salmivalli, 2017). The success of teacher intervention has important implications in terms of how students should be effectively supported, and how their confidence and sense of security might increase. Trying to deepen factors predicting a successful response of teachers to bullying is a priority to define the most important components for teachers’ training (Yoon et al., 2011; Gregus et al., 2017).

The current study aims to consider all these associations in a complex model where teachers individual factors foster an effective responding to bullying situations, which in turn is associated with a lower students’ perception of school bullying. Both the perspectives of teachers and students will be included in the study: the teachers’ perception of their individual and professional characteristics and their likelihood to respond to a bullying episode, and the students’ perception of school level of bullying and victimization.

### Teachers Responding (or Not) to a Bullying Episode

Teacher’s responses to bullying vary considerably, including different strategies focused on the victim, on the bullies, or on the group. Seidel and Oertel (2017) specifically distinguished three different strategies used by the teachers. First, they list authoritarian-punitive strategies (i.e., threats, discipline, expulsion) that are among the most used by teachers (Bauman et al., 2008; Sairanen and Pfeffer, 2011; Burger et al., 2015). However, they have only a minimal effect on successful interventions with students, because no positive model for social behavior modification is proposed. Another strategy used by teachers is individual assistance directed to the victims and the bullies, supporting them emotionally, and increasing empathy toward students who have been victimized (Bowes et al., 2010; Ledwell and King, 2015; Menesini and Salmivalli, 2017). The third strategy includes the supportive-cooperative intervention, which involves all the students in the class in order to promote cooperation among students and to define actions at class and/or school level with the support of parents and other professionals (Seidel and Oertel, 2017).

Sometimes teachers do not intervene (Yoon and Kerber, 2003; Bauman and Del Rio, 2006; Yoon et al., 2016; Divecha and Brackett, 2019) and the reasons for this may vary. They may simply be unware of the bullying phenomenon (Smith and Shu, 2000; Fekkes et al., 2005; Bauman and Del Rio, 2006). Individual differences in teachers’ beliefs and attitudes will influence if and how they respond to instances of school bullying (Yoon and Kerber, 2003; Kochenderfer-Ladd and Pelletier, 2008; Veenstra et al., 2014). Some teachers consider bullying to be a normative behavior that may help children to acquire social norms (Kochenderfer-Ladd and Pelletier, 2008) and find it unnecessary to intervene. In other cases, they do not intervene because they do not feel sympathy for the victim (Yoon and Kerber, 2003). Besides, teachers are unlikely to intervene in bullying situations when they feel they could not obtain any results (Dedousis-Wallace et al., 2013), when they perceive the behavior is not bullying, or when more hidden forms such as relational or verbal bullying are occurring (Blain-Arcaro et al., 2012; Duy, 2013; Haig et al., 2013), because they are often not perceived as bullying by teachers (Bilz et al., 2016).

If teachers ignore or trivialize bullying, or if students interpret teachers’ lack of intervention as an implicit acceptance of bullying, it is more likely that aggressive behavior will increase (Huesmann et al., 1984; Burger et al., 2015; Wachs et al., 2016). The students who have been victimized can be discouraged from reporting bullying incidents in the future, and the students who observed the bullying can feel less motivated to intervene or ask for help (Huesmann et al., 1984; Burger et al., 2015; Wachs et al., 2019). When teachers intervene and make an end to the situation of bullying, they communicate that bullying is not acceptable, and consequently students are less inclined to justify this type of behavior (Campaert et al., 2017). Also, by intervening personally, the teacher communicates that no justifications are acceptable in school (Veenstra et al., 2014; Oldenburg et al., 2015; Saarento et al., 2015). On the other hand, teachers’ nonintervention tends to justify this behavior, resulting in the students classifying it as normal (Campaert et al., 2017).

### Factors Predicting Teachers’ Response to a Bullying Situation

The existing literature (Yoon and Bauman, 2014; Troop-Gordon and Ladd, 2015) underlined that teachers’ individual and professional variables (i.e., the teacher’s attitudes, perception of efficacy, beliefs and knowledge, the level of empathy); relational variables (i.e., the quality of the teacher-student relationship); and their interaction with more contextual and situational factors (i.e., class climate, school liking, bullying characteristics) are associated with the likelihood that bullying and victimization can occur. In the current study, we will focus our attention on teachers’ individual factors.

Among those, teachers’ self-efficacy assumes a central role. Literature showed that teachers with higher self-efficacy are more likely to intervene both for direct and indirect forms of bullying (Fischer and Bilz, 2019). Overall, higher levels of teachers’ self-efficacy increase the likelihood to identify victims and to understand the victims’ sufferance (Oldenburg et al., 2015; Nappa et al., 2018), increase the efforts teachers put in the intervention, and the success of those actions (Hawley and Williford, 2015). Several studies affirm that if teachers think that they are able to contribute to bullying decrease, they will intervene more often (Bradshaw et al., 2007; Duong and Bradshaw, 2013; Yoon and Bauman, 2014; Collier et al., 2015; Williford and Depaolis, 2016). Studies focused on the association between teachers’ self-efficacy and teachers’ response to bullying situation presented inconsistent findings. Some of them reported a significant and positive association between these two variables (Dedousis-Wallace et al., 2013; Collier et al., 2015), but others did not support the link (Yoon et al., 2016; Begotti et al., 2017). One explanation could be referred to the type of construct used, a general construct of teachers’ self-efficacy or a task-specific construct of teachers’ self-efficacy in bullying contexts (Bradshaw et al., 2007; Duong and Bradshaw, 2013; Yoon and Bauman, 2014). In some studies, teachers’ self-efficacy evaluates how confident they would feel when confronted with a particular hypothetical bullying situation (Dedousis-Wallace et al., 2013; Boulton et al., 2014; Collier et al., 2015), while other studies assessed teachers’confidence in handling general behavioral problems of their students (Yoon, 2004; Byers et al., 2011; Yoon et al., 2016; Begotti et al., 2017). Thus, the role of the perceived self-efficacy of teachers as predictor of teachers’ intervention when an episode of bullying occurred is not clear. More attention should be paid to the domain-specific or general nature of self-efficacy construct.

Another variable to understand personal and professional predictors of teachers’ behavior is job satisfaction. Teachers’ job satisfaction is a multifaceted construct (Herzberg, 1959) that regards the positive or negative evaluative judgment that people make about their job. Past research indicated that teachers’ job satisfaction is related to a range of positive outcomes, including job performance (Judge et al., 2001), enthusiasm (Weiqi, 2008), commitment (Reyes and Shin, 1995), and attitudes toward their daily work (Caprara et al., 2003a,b). Teachers can attain job satisfaction while performing daily teaching activities such as working with students, monitoring students’ learning progress, working with colleagues, and contributing to an inclusive school climate (Cockburn and Haydn, 2004). Previous research revealed also significant positive relationships between teachers’ self-efficacy and job satisfaction (Bogler, 2001). Job satisfaction is very likely to be associated with teachers’ self-efficacy and helps to support efforts toward the achievement of optimal academic outcomes for students (Caprara et al., 2006). Teachers with a high level of self-efficacy are more likely to manage certain situations and to promote interpersonal networks that consolidate and support their job satisfaction (Caprara et al., 2006). The sense of perceived competence is a primary resource for intrinsic motivation and satisfaction. In the case of teachers, job satisfaction is related to self-efficacy both with respect to the profession itself and to the environment in which they work. The research carried out indicates that although teachers are generally satisfied with aspects of their professional life related to teaching, they tend to be dissatisfied with the aspects concerning the performance of their work. As a consequence, higher levels of satisfaction correspond to greater commitment and better performance. The level of satisfaction has also a growing influence on teachers’ attitudes and efforts in the implementation of daily activities (Caprara et al., 2003a,b). To our knowledge, no studies have yet analyzed the impact of job satisfaction on teachers’ interventions in case of bullying and victimization.

Besides, the specific competence in relation to the phenomenon that teachers might have can influence the likelihood of teachers’ intervention and in turn the level of bullying in schools. Literature highlights how teachers who have specifically dealt with the issues of bullying and who actively participate in prevention projects are perceived to be more effective and confident in handling victimization problems have more supportive attitudes toward victims and feel safer in working with families on these problems. These aspects are positively correlated to a decrease in the phenomenon (Alsaker, 2004). According to this view, Veenstra et al. (2014) found that classrooms with teachers that were not perceived by their students as competent in reducing bullying displayed a higher level of peer victimization. We hypothesized that teachers who feel competent will be more able to actively deal with the bullying issue in their school, than those who feel incompetent or indifferent, who could be more passive observers of students dynamics.

### Objective and Hypothesized Model

**Figure 1:**
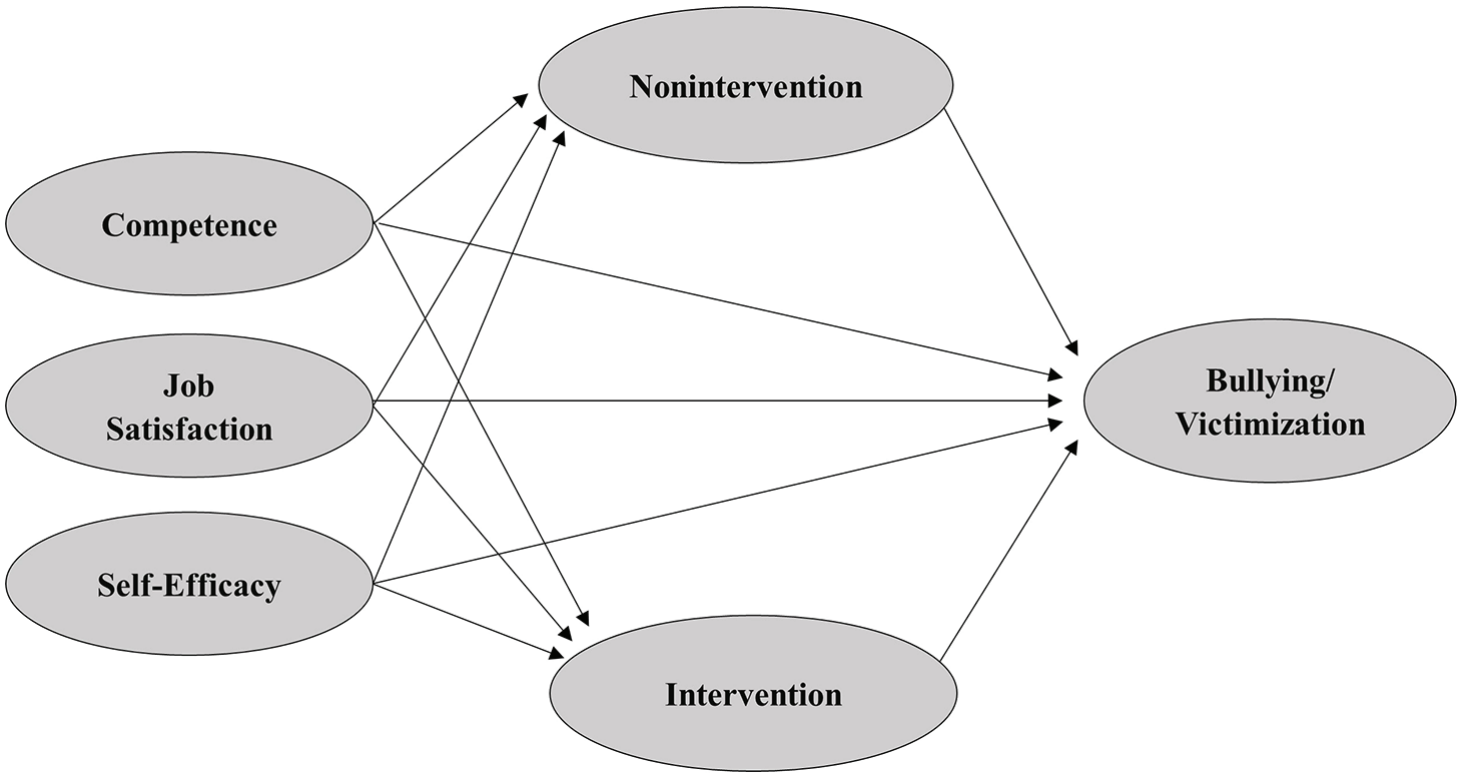
Hypothesized model.

Starting from these considerations, the current study aims to evaluate the impact of teachers’ individual factors on the students’ perception of bullying and victimization through the teachers responding to a bullying episode. In particular, we hypothesize that the teachers’ competence regarding the phenomenon, job satisfaction, and self-efficacy will be negatively linked to the level of school bullying and victimization and that teacher intervention or nonintervention will mediate the association between teachers’ individual variables and school bullying and victimization. According to a social-cognitive model, we hypothesize that if teachers feel themselves as more competent in addressing bullying, more satisfied with their work, and more self-efficacious, they would intervene more frequently and with better results and therefore students would report lower levels of bullying and victimization at school. On the contrary, when teachers feel less competent, less satisfied with their work, and less self-effective, there is a higher probability of teachers not intervening, and this in turn can be related to higher levels of bullying and victimization (Figure 1).

## Materials and Methods

### Participants and Procedure

Participants to this study included students and their teachers enrolled in the NoTrap! program, a prevention program of bullying and cyberbullying carried out in Italy (Palladino et al., 2016). In particular, the current study considered the first data collected in 2017 (pre-intervention). The sample consisted in teachers and their students enrolled in grades 7 through 9 in Tuscany (Provinces of Lucca, Florence and Pistoia). The study was conducted with 120 teachers (17.5% boys; 79.2% girls), between ages 25 and 66 (mean age = 48.21; SD = 9.22), and 1,056 students (40.3% boys; 59.6% girls), between ages 11 and 17 (mean age = 13.09; SD = 1.46). Of these, 57% attended first level secondary school and 42.2% attended high school.

The project considered the involvement of referent teachers for each school. A total of 56 teachers in the province of Florence, 31 teachers in the province of Lucca, and 33 teachers in the province of Pistoia participated in the training. The number of teachers per school ranged from 3 to 30: this variability can be explained considering the number of classes involved in the project in each school, the number of students per school, and finally the number of teachers already trained in each school. All the teachers were invited to participate in two training sessions held by the staff of the University of Florence. During the first meeting, a questionnaire was administered to the teachers.

The research was carried out in accordance with the recommendations of the Italian Association of Psychology. The research project was approved by the school committees and the heads of the school based on school standards. Parents’ active consent was obtained prior to questionnaire administration. Parents and students were informed about the confidentiality and anonymity of their responses, that their participation was entirely voluntary, and that they could withdraw at any time. The informed consent procedure consisted of the preliminary approval by the school principal and the class council. Once the school gave its permission, a letter was sent to all the students and their parents, informing them of the project and asking them to complete and turn in the permission slip to participate. Ninety-six percent of the target sample received active consent from parents to participate in the project and intervention.

### Measures

#### Teacher’s Competence

The *ad hoc* questionnaire measuring the level of competence and knowledge of bullying is composed by two items (“How competent do you feel about bullying issues?”; “How competent do you feel about cyberbullying issues?”) rated on a 4-point Likert-type scale ranging from 0 (“not at all”) to 3 (“very much”). The Cronbach’s alpha was 0.83 and the inter-item correlation was 0.71.

#### Job Satisfaction

A short version of the Italian version (Borgogni et al., 2010) of the *Job Descriptive Index* (Smith et al., 1969) was used for the measurement of teacher satisfaction with their job. Five statements (“I feel good at work”; “I feel satisfied with what I reach at work”; “I am happy with the way my superiors treat me”; “I am satisfied with my work”; “I am happy with the way my colleagues treat me”) rated the construct on a 7-point Likert-type scale (0 = absolutely disagree; 7 = absolutely in agreement). The Cronbach’s alpha was 0.86.

#### Teachers’ Self-Efficacy Beliefs

The scale consisted of five items rated on a 7-point Likert-type scale (0 = absolutely disagree; 7 = absolutely in agreement) (Caprara et al., 2006) evaluating teacher’s perceived capability: (1) to cope with didactical tasks (e.g., “I am capable of overcoming all the challenges I encounter in meeting my teaching objectives”); (2) to handle discipline problems in the class (e.g., “I can make my students respect rules and codes of conduct”); (3) to earn appreciation from colleagues and families (e.g., “I am able to earn the trust and appreciation of all my colleagues”); and (4) to take advantage of innovations and technologies to better their work (e.g., “I am capable of taking full advantage of technological innovations in my teaching”). The Cronbach’s alpha was 0.84.

#### Teachers’ Intervention and Nonintervention After a Bullying Situation

In order to evaluate teachers’ interventions in incidents of bullying and victimization, we used a revised version of the Teachers’ responses to incidents of bullying and victimization (Yoon and Kerber, 2003; Bauman and Del Rio, 2006; Campaert et al., 2017). The questionnaire asked teachers to fill out a scale listing different types of teachers’ behavioral reactions and asked them to rate how often they responded with the proposed strategies. The scale consisted of three items measuring nonintervention (“does not intervene,” “leaves things up to the students,” and “is not aware about it”), and five items measuring intervention ranging from mediation, group discussion, victim support, and disciplinary sanction for the bully (“Help the boys involved to resolve the conflict”; “I discuss the episode with the whole class”; “I try to help the victim”; “I take measures against the bully”), rated on a 5-point Likert-type scale from 0 (never) to 4 (daily). The Cronbach’s alphas were 0.85 for intervention and 0.90 for nonintervention. We derived a measure of teacher intervention and nonintervention in bullying/victimization situation.

#### Bullying and Victimization

The Florence Bullying and Victimization scales (FBVSs; Menesini et al., 2011; Palladino et al., 2016) were used, consisting of two subscales: one for bullying and the other for victimization. The FBVSs consist of 20 items, investigating the frequency with which adolescents have perpetrated or have experienced bullying in the two previous months. The answers were assessed on a 5-point Likert scale from “never” to “several times a week.” The two subscales were composed of 10 items each. The reliability coefficients showed good values: for bullying we had a Cronbach alpha of 0.79 at T1 and for victimization, a Cronbach alpha of 0.84.

### Data Analyses

The data were analyzed starting from the teacher data. Individual teachers’ variables were associated with the school level of bullying and victimization. The school level of bullying and victimization was defined calculating the school-level means reported by the students.

Preliminary analyses were conducted to examine the correlations between the teachers’ self-efficacy, competence, job satisfaction, intervention and nonintervention, and bullying and victimization. Two path analysis models were used to test the proposed direct and indirect models, one for bullying and the other for victimization. The models tested whether teacher’s competence regarding bullying, job satisfaction, and self-efficacy were linked to level of bullying and victimization through levels of teacher intervention and nonintervention after a bullying situation.

All the analyses were conducted *via* Mplus 4.0 (Muthén and Muthén, 1998–2012). All models estimated direct and indirect paths. The significance of the indirect paths was analyzed by the test of the indirect effect in Mplus (Muthén and Muthén, 1998–2012).

## Results

Table 1 reported bivariate correlations, means, and standard deviations for the variables considered. As we can see, the perception of competence on bullying, self-efficacy, and job satisfaction are all intercorrelated, in particular self-efficacy and job satisfaction. Besides, bullying and victimization behaviors are associated with satisfaction and with teachers’ intervention.

**Table 1:** Correlations among study variables, means, and standard deviations.

	1	2	3	4	5	6	7	Mean (SD)
1. Competence	1							2.22 (1.14)
2. Satisfaction	0.250^**^	1						5.88 (0.90)
3. Self-efficacy	0.233^*^	0.544^**^	1					5.25 (0.09)
4. Intervention	0.327^**^	0.170	0.173	1				3.93 (0.26)
5. Nonintervention	−0.174	−0.219^*^	−0.181	0.033	1			1.74 (0.64)
6. Bullying	−0.063	−0.210^*^	−0.108	−0.230^*^	0.051	1		1.06 (0.13)
7. Victimization	−0.024	−0.194^*^	−0.082	−0.094	−0.077	0.985^**^	1	1.07 (0.13)

* p < 0.05;

** p < 0.01;

*** *p < 0.001*.

For the model predicting bullying, findings showed a positive and significant effect of teacher’s competence on his/her intervention, which in turn is negatively associated with bullying. This indirect effect through teachers’ intervention resulted significant (*β* = −0.079, SE = 0.028, *p* = 0.004).

**Figure 2:**
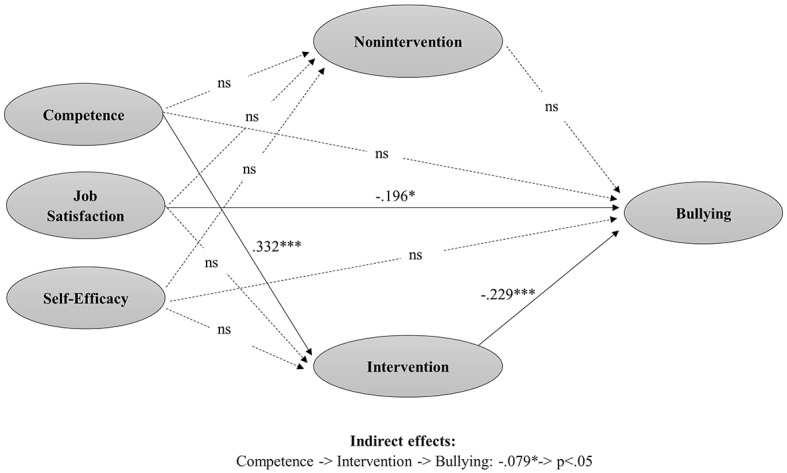
Path analyses of obtained relations among teacher’s competence regarding bullying management, job satisfaction, self-efficacy, teacher’s intervention, teacher’s nonintervention, and level of bullying.

Higher levels of teachers’ competence influence a higher likelihood that teachers intervene after a bullying situation (*β* = 0.332, SE = 0.086, *p* < 0.001), which in turn influences lower levels of bullying (*β* = −0.229, SE = 0.047, *p* < 0.001). A direct and negative effect of job satisfaction on bullying was also found (*β* = −0.196, SE = 0.113, *p* = 0.019). No significant path has been found for nonintervention (Figure 2; Table 2).

**Table 2:** Estimated regression coefficients in the model of bullying.

Criterion	Predictors	*Β*	SE	*p*	*R*^2^
Bullying	Competence	0.065	0.107	0.566	
	Self-efficacy	0.028	0.084	0.797	
	Job satisfaction	−0.196	0.113	0.019	
	Nonintervention	0.028	0.089	0.753	
	Intervention	−0.229	0.047	<0.001	0.089
Nonintervention	Competence	−0.105	0.090	0.243	
	Self-efficacy	−0.075	0.119	0.532	
	Job satisfaction	−0.161	0.124	0.196	0.067
Intervention	Competence	0.332	0.086	<0.001	
	Self-efficacy	0.070	0.106	0.510	
	Job satisfaction	0.049	0.113	0.668	0.140

**Figure 3:**
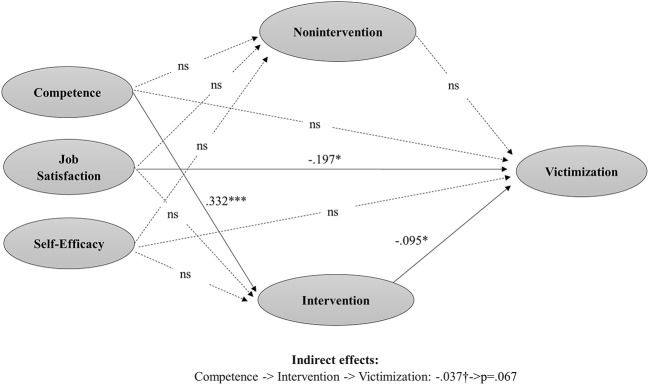
Path analyses of obtained relations among teacher’s competence regarding bullying management, job satisfaction, self-efficacy, teacher’s intervention, teacher’s nonintervention, and level of victimization.

For the model predicting victimization, findings are quite similar, except for the indirect effect which is now not significant. A positive and significant effect was found for competence on teacher’s intervention (*β* = 0.332, SE = 0.086, *p* < 0.001), which in turn is negatively associated with victimization (*β* = −0.095, SE = 0.048, *p* < 0.05). However, the indirect effect through teachers’ intervention resulted not significant in this case (*β* = −0.037, SE = 0.020, *p* = 0.067). A direct and negative effect of job satisfaction on bullying was also found (*β* = −0.197, SE = 0.084, *p* = 0.020) (Figure 3; Table 3).

**Table 3:** Estimated regression coefficients in the model of victimization.

Criterion	Predictors	*Β*	SE	*p*	*R*^2^
Victimization	Competence	0.067	0.114	0.556	
	Self-efficacy	0.025	0.110	0.820	
	Job satisfaction	−0.197	0.084	0.020	
	Nonintervention	0.051	0.092	0.579	
	Intervention	−0.095	0.048	0.048	0.048
Nonintervention	Competence	−0.105	0.090	0.243	
	Self-efficacy	−0.075	0.119	0.532	
	Job satisfaction	−0.161	0.124	0.196	0.067
Intervention	Competence	0.332	0.086	<0.001	
	Self-efficacy	0.070	0.106	0.510	
	Job satisfaction	0.049	0.113	0.668	0.140

## Discussion

The current investigation examined the contribution of teachers’ competence, job satisfaction, and self-efficacy to the level of bullying and victimization in schools. In the first model explaining bullying, teacher’s competence regarding bullying was associated to the level of bullying through its effect on teacher’s intervention. If the teachers feel themselves as competent about bullying, they intervene more frequently with positive strategies and this is consequently associated to decrease of class bullying. On the other side, higher teachers’ job satisfaction directly influences lower levels of bullying. No specific effect has been identified regarding teachers’ self-efficacy. The same model resulted for predicting the level of victimization, except for the non-significance of the indirect effect.

Teachers who perceived themselves as more competent in the bullying phenomenon are more prone to intervene in cases of bullying and victimization. Competence can be fostered through specific trainings aimed to define the phenomenon, to underline the dynamics of the problem, and to present the best intervention strategies. This finding supports previous literature (Alsaker, 2004), where teachers who have more extensive knowledge of the phenomenon are more effective in managing problems, they have more supportive attitudes toward the victims, and are perceived to be more effective and confident in handling episodes of bullying.

On the other side, the finding that teachers’ self-efficacy was not associated with a more proactive and effective role of teachers in handling bullying is in contrast with some of the previous studies (Dedousis-Wallace et al., 2013; Collier et al., 2015). Two main explanations can be considered for this result. First, the teachers’ competences and self-efficacy were highly correlated. When considered together, the contribution of specific knowledge and competences on bullying was more relevant as compared to the general teachers’ self-efficacy. Secondly, the construct of teachers’ competence was specific for the bullying content, whereas the construct of teachers’ self-efficacy was not. In the literature we find that a task specific construct of self-efficacy is associated to bullying and not a general construct of professional self-efficacy as the one we tested in our study (Bradshaw et al., 2007; Duong and Bradshaw, 2013; Yoon and Bauman, 2014).

The role of job satisfaction is important for class bullying reduction. Job satisfaction includes different aspects such as the class climate, satisfaction for the work done, and the quality of relations with the other teachers. The hypothesis explains the direct relationship between satisfaction and both bullying and victimization and expresses the concept that being satisfied with one’s work can directly influence the attitude teacher has toward his/her students. This can enhance a more positive classroom climate, better interpersonal relationships, and greater collaboration between students and teachers, and finally, it can directly influence the level of both bullying and victimization within the class. Teachers’ satisfaction can be perceived by the students in their everyday life in school because it constantly influences the quality of interactions and relationships in the classroom.

In disagreement with the literature (Campaert et al., 2017) and with our hypothesis, a relationship between nonintervention and bullying and victimization levels was not found. In particular, the nonintervention does not appear to be a mediator between teachers’ predictors and the level of bullying and victimization. The result could probably be due to the different evaluation sources used in this study compared to the studies carried out by Campaert et al. (2017). We considered two different perceptions: the self-evaluation of the teacher for the individual factors and the level of intervention and nonintervention, and the students’ evaluation of the level of school bullying. In addition, we used a model testing the intervention and not the nonintervention. In fact, teacher’s variables used were assumed to be predictors of the intervention. Further studies could compare both perspectives, i.e., the perceptions of teachers and students, deepening the relation between the two and how they can explain teachers’ interventions.

### Implications for Intervention

Current findings have relevant implications in terms of designing interventions for bullying prevention involving teachers. In particular, the study suggested relevant guidelines for defining which components should be implemented in teachers’ training.

Raising awareness on this phenomenon is the first step, along with promoting knowledge about bullying and victimization. Second, increasing skills and competences on the effective way to intervene after a bullying episode seems to be crucial. Besides, supporting the teachers’ experience and monitoring the process may be relevant in order to develop a true sense of self-efficacy in teachers who are dealing with bullying and victimization. According to Bandura, in order to foster self-efficacy, teachers need opportunities to practice bullying intervention skills, to implement specific strategies, to observe successful interventions by others, and to be exposed to positive prevention messages (Bandura, 1994). Confronting with direct and effective experiences, sharing best practices, implementing procedures for responding to bullying situation “in a safe condition” allows teachers to adapt their skills and strategies in a positive way. This opportunity to gain direct experience is a resource for the teacher to feel more aware and confident about what to do if bullying occurs in class.

Third, job satisfaction resulted a key variable for the daily work in class. Job satisfaction is associated with teachers’ self-efficacy (Caprara et al., 2006) and the sense of perceived competence. Both are a primary resource for intrinsic motivation and satisfaction. To improve job satisfaction, interventions should maximize principal’s support, affiliation among staff members, and goal consensus toward a common school policy.

### Limitation and Future Studies

This study has several limitations. Among them, we can highlight the voluntary nature of the participation of teachers to the training and the consistent variability of the number of teachers involved per school. A second limitation is the fact that the analysis was done at school level and not at a class level. This is because the teachers’ questionniares were anonymous. We collected information about the school but not about the class to respect their anonymity. The cross-sectional nature of the study did not allow to define causal paths. Teachers’ job satisfaction was investigated only as a predictor of bullying. In future research, a longitudinal design could be used to understand if job satisfaction could be a predictor or a result of students and school climate or could be explained by a circular relationship between job satisfaction and quality of school climate and level of bullying. Finally, the measure of competence on bullying was only composed by two items. Future studies should include a stronger and validated measure of teachers’ competence on bullying.

## Data Availability

The datasets for this manuscript are not publicly available because we did not request an explicit consent to school and to the parents for the public availability of the data.

## Ethics Statement

The research was carried out in accordance with the Ethic Research Recommendations of the Italian Association of Psychology. It was not approved by a Ethical Research Committee because at that time (2016) the University of Florence did not have an Ethical Committee, neither the approval was specifically requested by the Italian Association of Psychology. Initially, the research project was approved by the school committee and the Head of the School who considered the study eligible for the school standards. Students or parents’ written consent was obtained following the country law. Specifically, in Italy school’, parents’ and students’ active consent were obtained prior to conduct the questionnaires administration. Participation was entirely voluntary, confidential and anonymous. Participants were all informed that they could withdraw from the study at any time. We confirm that 4% of participants who did not have parental consent were excluded from the study.

## Author Contributions

All the three authors listed have contributed sufficiently to the project to be included as authors, and all those who are qualified to be authors are listed in the author byline.

### Conflict of Interest Statement

The authors declare that the research was conducted in the absence of any commercial or financial relationships that could be construed as a potential conflict of interest.
